# Retinal organoids: a window into human retinal development

**DOI:** 10.1242/dev.189746

**Published:** 2020-12-24

**Authors:** Michelle O'Hara-Wright, Anai Gonzalez-Cordero

**Affiliations:** 1 Stem Cell Medicine Group, Children's Medical Research Institute, University of Sydney, Westmead, 2145, NSW, Australia; 2 School of Medical Sciences, Faculty of Medicine and Health, University of Sydney, Westmead, 2145, NSW, Australia

**Keywords:** Retinal organoids, Stem cells, Human development

## Abstract

Retinal development and maturation are orchestrated by a series of interacting signalling networks that drive the morphogenetic transformation of the anterior developing brain. Studies in model organisms continue to elucidate these complex series of events. However, the human retina shows many differences from that of other organisms and the investigation of human eye development now benefits from stem cell-derived organoids. Retinal differentiation methods have progressed from simple 2D adherent cultures to self-organising micro-physiological systems. As models of development, these have collectively offered new insights into the previously unexplored early development of the human retina and informed our knowledge of the key cell fate decisions that govern the specification of light-sensitive photoreceptors. Although the developmental trajectories of other retinal cell types remain more elusive, the collation of omics datasets, combined with advanced culture methodology, will enable modelling of the intricate process of human retinogenesis and retinal disease *in vitro*.

## Abstract (precis)

**Summary:** Retinal organoid systems derived from human pluripotent stem cells are micro-physiological systems that offer new insights into previously unexplored human retina development.

## Introduction

Retinogenesis is the formation of the retinal lamellae that comprise seven retinal cell types. During vertebrate neurulation, the forebrain divides to form two secondary brain vesicles: the telencephalon and diencephalon. In the diencephalon, the eye field region is first partitioned into a pair of optic vesicles, the precursors of the optic cups that give rise to the retinal pigment epithelium (RPE) and the neural retina (NR) (Müller and O'Rahilly, 1985; Pearson, 1980; Perron et al., 1998; Zuber et al., 2003).

**Fig. 1.**
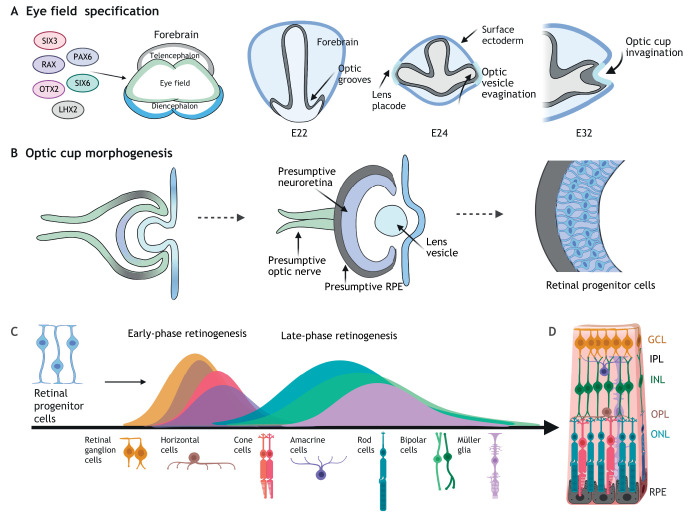
**Building the retina: eye field specification, optic cup morphogenesis and retinal cell differentiation.** (A) *In vivo*, the eye field transcription factors SIX3, RAX, PAX6, OTX2, SIX6 and LHX2 specify the presumptive eye field in the diencephalon of the developing forebrain. By human embryonic day 22 (E22), indents (optic grooves) form in the neural fold, bilaterally to the diencephalon. The optic grooves evaginate by E24 to form the optic vesicles, which protrude to contact the overlying surface ectoderm at the site of the presumptive lens (lens placode). Invagination of the optic vesicle forms the bilayered optic cup by E32. (B) The inner layer of the optic cup specifies the presumptive neuroretina, whereas the outer layer specifies the presumptive retinal pigment epithelium (RPE). The optic vesicles remain connected to the forebrain via the optic stalk, a hollow connection that closes to form the presumptive optic nerve. The lens vesicle pinches off the surface ectoderm. In the presumptive neuroretina, multipotent retinal progenitor cells (RPCs) begin to differentiate into retinal cell types. (C) Differentiation of the seven main retinal cell types from RPCs proceeds sequentially in waves, with retinal ganglion cells, horizontal cells, cone photoreceptor cells and amacrine cells formed in an early retinogenesis wave, followed by the overlapping late-phase generation of rod photoreceptor, bipolar and Müller glia cells. (D) These cell types populate the multi-layered retina from the basal-most ganglion cell layer (GCL), inner plexiform layer (IPL), inner nuclear layer (INL), outer plexiform layer (OPL) and apical-most outer nuclear layer (ONL), where photoreceptors lie adjacent to the RPE.

Studies in model organisms have informed the molecular basis of eye formation. Eye field transcription factors (EFTFs) *PAX6*, *RAX*, *SIX3*, *LHX2*, *SIX6* and *OTX2* specify the presumptive eye field and optic groove formation (Zuber et al., 2003). Through protrusion into the surrounding mesenchyme, the optic vesicle contacts the overlying surface ectoderm, before it invaginates to form the double-walled optic cup (Fig. 1A). The inner and outer walls of the optic cup will form the RPE and the NR, respectively. The presumptive optic nerve forms from a hollow primitive optic stalk connecting to the forebrain (Fig. 1B) (reviewed by Adler and Canto-Soler, 2007; Chow and Lang, 2001; Fuhrmann, 2010).

Multipotent retinal progenitor cells (RPCs) undergo division to specify and differentiate retinal cells in a sequential manner, according to the competence model of differentiation (Cepko et al., 1996). Retinal ganglion cells (RGCs), cone photoreceptor cells, and horizontal and amacrine cells are generated in the early phase, overlapping with the late-phase generation of rod photoreceptors, bipolar cells and Müller glia (Cepko et al., 1996) (Fig. 1C). RPCs populate the outer nuclear layer (ONL), with the cone and rod photoreceptor cells extending processes into the outer plexiform layer (OPL), where they form synapse networks with bipolar, horizontal and other interneurons in the inner nuclear layer (INL). These in turn synapse in the inner plexiform layer (IPL) with RGCs in the ganglion cell layer (GCL) (Fig. 1D) (Blanks et al., 1974; Fisher, 1979; Olney, 1968).

Studies of human eye development have been limited to the anatomical and morphological analysis of scarce human foetal tissue. With the emergence of pluripotent stem cells (PSCs) and organoid technology, the multiplicity of retinal development has been modelled in an organotypic 3D configuration, generating micro-physiologically active systems ‘in a dish’.

Much of the current research in the field has centred on applications of human retinal organoids for therapeutic and clinical implementation. Here, we will instead highlight the pertinence of organoids to model human eye development in a temporal and spatial context. The capacity to generate large-scale transcriptomic datasets has offered new insights into human retinogenesis. Recent studies have both uncovered previously unknown developmental networks and trajectories, and, via comparison with their *in vivo* counterparts, have offered validation and assessment of the authenticity at which we are currently able to recapitulate human retinogenesis *in vitro.*

In this Review, we provide a brief overview of eye development and the evolution of PSC studies toward 3D cultures of retinal tissue. We also summarise the current understanding of human eye development revealed from human (h)PSC-derived retinal organoids and pinpoint the caveats of this model that must be addressed in future studies.

## Orchestrating human eye development

Human eye development was first characterised by O'Rahilly and Müller's description of the human Carnegie stages of development (Müller and O'Rahilly, 1985; O'Rahilly and Müller, 2010). On human foetal embryonic day 22 (E22), optic grooves form bilaterally to the diencephalon, evaginating to form optic vesicles by E24: Carnegie stage 11 (Fig. 1A) (Müller and O'Rahilly, 1985).

Early model organism studies employing genetic mutants, embryonic manipulation and retinal explants alluded to crucial molecular components implicated in ocular and retinal development, revealing the complex interplay of signalling networks and multifaceted cell-cell interactions guiding early eye development. However, compared with humans, these models differ in cellular composition, morphology and ocular function (Gibson, 1938; Uga and Smelser, 1973). Therefore, stem cell-derived models of human retinal development may provide a relevant alternative to complement animal studies.

## The beginnings of stem cell-derived retinal cultures

**Fig. 2.**
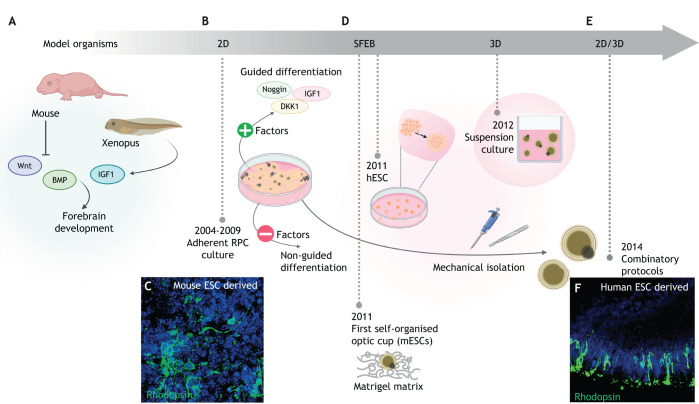
**The journey from classical developmental biology to three-dimensional organoid models of retinogenesis.** (A) Model organism studies identified basic molecular drivers of retinogenesis, with key studies finding that inhibition of Wnt and BMP signalling in the mouse or injection of IGF1 into *Xenopus* embryos, induces forebrain development. (B) On this basis, early methods of 2D stem cell retinal differentiation incorporated Wnt or BMP inhibitors (DKK1 and Noggin, respectively) and/or exogenous IGF1 in a ‘guided’ approach, before non-guided spontaneous approaches emerged. Adherent culture of retinal progenitor cells (RPCs) was first demonstrated in the early 2000s. (C) Adherent cultures demonstrated the *in vitro* generation of photoreceptors [mouse embryonic stem cell (mESC)-derived rhodopsin^+^ photoreceptors cells in green], but these lacked lamellar organisation. (D) Eiraku et al. (2011) first demonstrated spontaneous generation of 3D optic cups from mESCs, enabling the self-organisation of retinal lamella with the addition of Matrigel matrix using a serum-free floating culture of embryoid body (EB)-like aggregates (SFEB) method. Subsequently, SFEB methods were used to generate retinal vesicles from human embryonic stem cells (hESCs), before multiple groups began to generate retinal organoids in true 3D suspension culture or (E) in combinatory 2D/3D approaches. In the latter, retinal vesicles spontaneously form from confluent cultures of PSCs and are mechanically excised from adherent culture before being placed into suspension culture. (F) In contrast to early 2D adherent cultures, this facilitated the organisation of rhodopsin (green)-expressing photoreceptors in a defined presumptive ONL. (C) Reproduced, with permission, from West et al. (2012). (F) Reproduced from Gonzalez-Cordero et al. (2017) where it was published under a CC-BY 4.0 license.

Classical developmental biology inferred the molecular basis of eye development to begin generating retinal cell types from PSCs. As BMP and Wnt antagonism is crucial for forebrain induction in *Xenopus* and mice, early 2D differentiation protocols used exogenous expression of the Wnt antagonist DKK1 and the BMP antagonist Noggin to guide PSCs to an anterior neural fate (Banin et al., 2006; Glinka et al., 1997; Lamba et al., 2006; Mukhopadhyay et al., 2001). Given that ectopic eye formation occurs following injection of IGF1 mRNA into *Xenopus* embryos (Richard-Parpaillon et al., 2002; Pera et al., 2001), supplementation of IGF1 to hPSC-derived Noggin/Dkk1 *in vitro* cultures led to augmentation of retinal progenitor gene expression (Lamba et al., 2006) (Fig. 2A)*.* However, owing to absence of essential indirect or direct cell-cell communication, such as temporally controlled diffusible factors secreted by the RPE, 2D cultures did not truly recapitulate or promote the complex process of human retinogenesis (Fig. 2B,C).

## Evolving to 3D protocols: generating human retina in a dish

Sasai's landmark generation of a self-organised 3D optic cup and stratified neuroepithelia from mouse PSCs (mPSCs) paved the way for a new generation of retinal models, based on organoids that more closely replicate *in vivo* development (Eiraku et al., 2011). Using a modified version of the serum-free floating culture of embryoid body (SFEB)-like aggregates method, Eiraku et al. cultured mPSC-derived EBs in suspension under low-growth factor conditions with Matrigel to provide extracellular matrix (ECM). This induced spontaneous formation of Rax^+^ RPCs in optic vesicles, which invaginate into optic cup-like structures with proximal-distal patterning, thus specifying RPE and NR (Eiraku et al., 2011) (Fig. 2D). Invagination proceeds in an apically convex manner, reflecting an intrinsic capacity for biomechanical remodelling. This autonomous curvature drives formation of a wedge-shaped hinge epithelium, which mimics *in vivo* embryonic retinogenesis and is congruent with a relaxation-expansion model of self-organisation that may be modelled *in silico* (discussed by Eiraku et al., 2012).

Many groups have since adapted and optimised protocols to derive retinal organoids from hPSCs (Kuwahara et al., 2015; Lowe et al., 2016; Mellough et al., 2015; Meyer et al., 2011; Nakano et al., 2012; Singh et al., 2015; Wahlin et al., 2017). Evolving from earlier 2D studies, the first description of hPSC-derived organoids employed additional growth factors, Noggin and DKK1 to induce neural induction during early differentiation (Meyer et al., 2011). Other guided approaches incorporated IGF1, BMP4 or Wnt antagonists to induce NR (Kuwahara et al., 2015; Mellough et al., 2015; Singh et al., 2015). The bio-mechanical rules involved in mPSC-derived optic cup invagination also applied to hPSC-derived optic vesicles. This includes inactivation of myosins, motor proteins that mediate folding, wedge shaping of the hinge epithelium and the NR folding mechanism called tangential expansion (Nakano et al., 2012). This knowledge can only be learned from characterisation of early developmental events in PSC-derived 3D organoids.

A novel approach, exploiting both a 2D and 3D differentiation format, demonstrated spontaneous retinal induction and optic vesicle formation from confluent hPSCs, bypassing the aggregation step in SFEB methods and the requirement of Wnt/BMP antagonists (Reichman et al., 2014, 2017). This simple method of differentiation challenged the requirement for exogenous signalling molecules in retinal induction, instead relying on endogenous modulation of BMP and Wnt signalling. This tissue autonomy was also demonstrated in a non-confluent SFEB approach, whereby plating of early 3D aggregates onto laminin allowed formation of NR organoids (Zhong et al., 2014).

Irrespective of the inclusion or omission of growth factors, and specific adherent or suspension stages, retinal differentiation protocols universally involve a neural induction phase and isolation of the emerging neuroepithelia, followed by conditions to support early and late cellular maturation (Fig. 2E). Maturation of photoreceptors can be promoted by retinoic acid (RA) treatment in the pleiotropic all-trans-retinal form, whereas opsin-specific 9-cis-retinal enhances rod generation and hypoxia facilitates cell survival (Gonzalez-Cordero et al., 2017; Kaya et al., 2019; Wahlin et al., 2017; Zhong et al., 2014). The evolution of complementary differentiation protocols offers a variety of methods for generating retinal organoids that somewhat agree in developmental temporal timelines and with normal human retinogenesis. The concordance of developmental timelines represents a unique opportunity to model human eye development *in vitro.* However, caution should be exercised when considering the addition of exogenous factors and methods to accelerate development and maturation of cells. These forced approaches, albeit quicker and more affordable, alter the temporal timeline of human retinal development, possibly introducing artificial environments that may not faithfully replicate natural development. Below, we summarise insights provided by retinal organoid studies into the regulatory mechanisms of human retina development, from the early eye field to the development of a complete laminated retinal niche.

## A window into early retinal development

Retinal organoids have now enabled the molecular characterisation of early events in human eye development. EFTFs are expressed in most PSC-derived cultures within the first month, patterning the eye field-like regions that form optic vesicle structures (Zhong et al., 2014; Collin et al., 2019a,b). Nakano et al. described invagination and formation of optic cup structures, albeit at low efficiency, from hPSC-derived retinal organoids. This is observed from day 24 onwards (corresponding to Carnegie stage 14 in human embryos), later than in mPSC-derived organoids, replicating the typical species-specific schedule of morphogenesis (Nakano et al., 2012).

In the human foetus, surface ectoderm thickens at the site of optic vesicle interaction at E32, forming the lens placode and later the lens vesicle (Fig. 1A) (O'Rahilly and Müller, 2010; Pearson, 1980). In the developing mouse eye, optic cup invagination has been demonstrated after ablation of the lens (Hyer et al., 2003), challenging Spemann's classic experiments, in which ablation of the developing optic vesicle disrupted lens formation in the adjacent surface ectoderm (Spemann, 1901). The majority of differentiation protocols generating both mouse and human retinal organoids demonstrate optic vesicle formation in the absence of surface ectoderm and lens. However, optic cup formation is rare, suggesting that lack of other eye tissue, such as lens and/or cornea, or certain signalling pathways affects completion of morphogenesis *in vitro.*

**Fig. 3.**
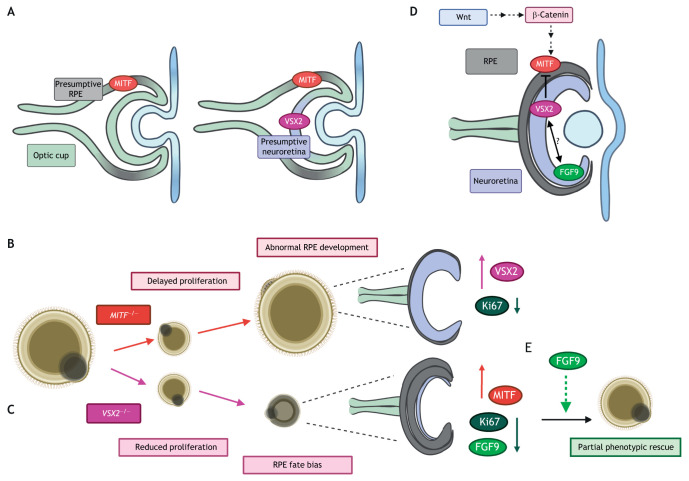
**Optic cup morphogenesis: a model of temporal inhibition and synergism.** (A) During optic cup formation and invagination, micropthalmia-associated transcription factor (MITF) and visual system homeobox 2 (VSX2) specify the presumptive retinal pigment epithelium (RPE) and neuroretina domain, respectively. MITF expression precedes VSX2 expression. (B,C) Studies using pluripotent stem cell (PSC)-derived *MITF*- and *VSX2*-mutant retinal organoids confirmed phenotypic findings. (B) *MITF*-mutant organoids exhibit delayed proliferation and downregulation of the proliferation marker Ki67 in early development, although long-term growth is unaffected. RPE develops abnormally and expression of the neuroretinal determinant VSX2 is upregulated. (C) *VSX2*-mutant organoids exhibit reduced proliferation in early development, followed by a fate bias towards RPE rather than neuroretina. Accordingly, downregulation of proliferation marker Ki67 and upregulation of RPE determinant *MITF* are apparent. (D) These studies also elucidated a novel model of VSX2 and MITF function and interaction in early retinogenesis. Before direct repression of MITF by VSX2 at the stage of determination of the neural retina and retinal pigment epithelium domains, MITF may play a role in proliferation during early development, potentially by acting downstream of the canonical Wnt/β-catenin pathway. (E) Exogenous expression of fibroblast growth factor 9 (FGF9) partially rescues the mutant phenotype, increasing expression of VSX2, but proliferation remains delayed. FGF9 may therefore work in concert with VSX2 to regulate early optic cup development (D).

Studies using patient-derived hPSCs have used optic vesicle phenotypes to uncover previously unknown mechanisms of early retinogenesis. hPSC-derived optic vesicles co-express visual system homeobox 2 (*VSX2*), the earliest specific NR marker, and microphthalmia-associated transcription factor (*MITF*), each of which becomes localised to the NR and RPE, respectively, at the optic cup stage (Capowski et al., 2014; Phillips et al., 2014) (Fig. 3A). This early pattern of commitment is in agreement with knowledge previously obtained in model organisms, particularly mouse, where *Vsx2* is expressed in RPCs until these reach a postmitotic state and eventually becomes restricted to bipolar cells (Belecky-Adams et al., 1997; Levine et al., 1994; Liu et al., 1994).

*VSX2* null mutations are associated with micropthalmia (abnormally small eyes) in humans and mice, with mice exhibiting drastically fewer retinal cells and no bipolar cells (Burmeister et al., 1996; Ferda Percin et al., 2000). Accordingly, retinal organoids derived from *VSX2* mutant patient-derived induced pluripotent stem cells (iPSCs) display impeded growth and fewer cells expressing the proliferation marker protein Ki-67 (Phillips et al., 2014). These cells also favour a phenotypic differentiation towards RPE over NR, with surviving NR regions lacking bipolar cells. *VSX2* mutant organoids showed upregulation of the RPE determinant transcription factor MITF and its downstream transcriptional targets, dopachrome tautomerase (*DCT*) and tyrosinase (*TYR*) (Phillips et al., 2014). *MITF* is expressed before *VSX2* and direct binding of VSX2 to the *MITF* promoter isoforms was identified in day 30 organoids via chromatin immunoprecipitation, thus offering an explanation of the RPE fate bias observed in *VSX2* mutant organoids (Capowski et al., 2014) (Fig. 3B,C).

These findings confirmed the well-established *VSX2-MITF* relationship in human-derived tissue. In mice, *MITF* repression by VSX2 in the presumptive NR orchestrates NR-RPE patterning (Bharti et al., 2012; Horsford et al., 2005; Nguyen and Arnheiter, 2000). Whereas MITF is restricted to the RPE and ciliary margin in normal development, *VSX2* mutant mice ectopically express MITF protein in the NR from E10.5 (Horsford et al., 2005).

*MITF* mutant hESC-derived organoids also contain significantly fewer Ki-67^+^ proliferative cells, with a significantly smaller diameter than isogenic controls (Capowski et al., 2014). However, unlike *VSX2* mutant organoids, in which photoreceptor maturation was delayed, long-term growth and photoreceptor marker expression was unaffected by reduced *MITF* expression (Capowski et al., 2014; Phillips et al., 2014). Thus, these studies in retinal organoids alluded to a proliferative role for MITF in early development at the stage preceding NR-RPE determination. MITF functions as a downstream effector of the canonical WNT/β-catenin pathway, which has established roles in dorsal-ventral patterning and optic cup morphogenesis, through regulatory feedback loops involving MITF and VSX2 (Bharti et al., 2012; Capowski et al., 2016; Cho and Cepko, 2006; Hägglund et al., 2013; Steinfeld et al., 2013). Accumulation of β-catenin in the dorsal optic vesicle specifies RPE, whereas low concentration of β-catenin in the ventral RPE causes trans-differentiation into NR (Fujimura et al., 2009; Hägglund et al., 2013; Liu et al., 2006; Westenskow et al., 2009). In the mouse, Wnt ligands derived from the surface ectoderm are also implicated in RPE differentiation (Carpenter et al., 2015). MITF has also been identified to directly interact with β-catenin, which is hypothesised to recruit β-catenin as a co-activator of MITF target genes (Schepsky et al., 2006) (Fig. 3D). Thus, MITF could potentially act in a similar way to expand the repertoire of canonical WNT signalling and exert pleiotropic effects in early human retinogenesis.

Fibroblast growth factors (FGFs) are abundantly expressed in ocular/extraocular tissues and have been identified as candidate surface ectoderm-secreted inducers of the NR (de Iongh and McAvoy, 1993; Nguyen and Arnheiter, 2000; Pittack et al., 1997). FGF3, FGF8, FGF9 and FGF19 are expressed at high levels in wild-type hESC-derived organoids (Phillips et al., 2014). Ectopic FGF expression in wild-type mouse optic vesicle cultures induces NR transformation and subsequent *Mitf* repression in the developing RPE, but not in *Vsx2* mutant cultures (Horsford et al., 2005). On this basis, it was hypothesised that FGF acts upstream of Vsx2 to mediate *Mitf* repression and specification of the NR. However, in early human-derived *VSX2* mutant organoids, addition of exogenous FGF9 only partially rescued disease phenotype, despite showing increased levels of VSX2, the phototransduction regulator recoverin and the bipolar marker calcium-binding protein 5 (CABP5) (Gamm et al., 2019) (Fig. 3E). FGF9 expression peaks in human organoids at day 10 and 20, representing periods of eye-field specification and optic vesicle formation, respectively (Gamm et al., 2019; Phillips et al., 2014). Inhibition of FGF signalling in early retinogenesis causes a similar phenotype to the *VSX2* mutation, but disruption of FGF or *VSX2* alone is not sufficient to prevent NR formation. Specifically, wild-type hPSC-derived organoids continue to form NR following FGF9 suppression (Gamm et al., 2019). Therefore, an alternative hypothesis is that FGF and VSX2 may act in concert in early human retinogenesis, rather than in series. This also highlights the existence of greater redundancy and plasticity in signalling pathways governing NR specification than initially deciphered from classical studies. Transcriptomic analysis of retinal organoids has identified this plasticity in the existence of cell cluster transition zones in early postmitotic cell fate specification between progenitors and differentiated neurons (Collin et al., 2019a; Cui et al., 2020; Sridhar et al., 2020). Signalling pathways and novel genes involved in RPC commitment were also identified in organoids, with single-cell RNA sequencing (scRNAseq) distinguishing two distinct RPC subtypes (Mao et al., 2019). Despite the ease of accessibility of early timepoints, few studies have focused on modelling early eye development using PSC-derived organoids. More insights into this area will further our understanding of retinogenesis, validating findings previously obtained in animal models.

## The development of cell types within retinal organoids

### The first born: retinal ganglion cells

**Fig. 4.**
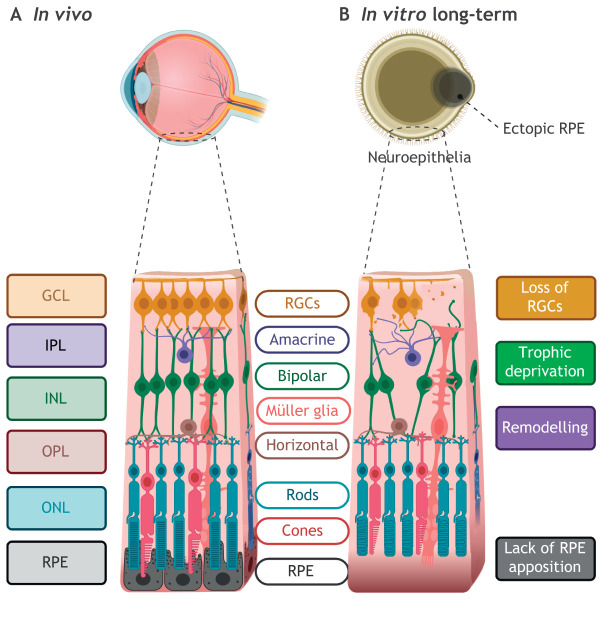
**Modelling retinal layers *in vitro*.** (A) *In vivo*, the seven main neuroretinal cell types populate the layers of the retina with retinal pigmented epithelium (RPE) next to the outer nuclear layer (ONL). Interneurons synapse with photoreceptors in the outer plexiform layer (OPL) and retinal ganglion cells (RGCs) in the inner plexiform layer (IPL) to relay signals to the brain. (B) *In vitro*, retinal organoids develop multiple layers and cell types, but RGCs are progressively lost in long-term culture, possibly owing to lack of neurotrophic factors or other ocular structures. Subsequently, interneuron cells are lost and remodelling occurs, possibly owing to trophic deprivation caused by loss of synaptic partner RGCs. In retinal organoids, RPE, a major source of diffusible factors, forms in adjacent clumps rather than juxtaposed to the ONL.

RGCs, the first cell type generated *in vivo*, are the retinal neuronal outputs, connecting to the brain through the optic nerve (Cepko et al., 1996; Rapaport et al., 2004; Young, 1985). The initiation, outgrowth and innervation of the axonal projections with the optic nerve requires complex signalling programme. Graded expression of transcription factors and chemoattractive or chemorepellent molecules, combined with intracellular signalling, mediate this development in animal models (Drescher et al., 1995; Monnier et al., 2002; Sakurai et al., 2002; Sakuta et al., 2001; Wahl et al., 2000; Xiang, 1998). RGCs form in retinal organoids but are stochastically and progressively lost in long-term cultures (Fig. 4) (Zhong et al., 2014). *In vitro*, typical RGC markers POU4F1 and NEFL are expressed at lower levels than in foetal samples (Sridhar et al., 2020). In scRNAseq studies, RGC-related genes, including those implicated in axon guidance, are responsible for the largest disparity between human foetal and organoid datasets, even at early time points (Brooks et al., 2019; Kaya et al., 2019; Sridhar et al., 2020).

Considering most retinal organoid differentiation protocols were manipulated to enrich for photoreceptors, it might be expected that conditions are non-optimal for deriving RGCs. However, to allow the investigation of the interaction of RGCs and other interneurons, and model retinogenesis as a whole, retinal organoids need to sustain RGCs. RGC neurite outgrowth is promoted via substrate modulation, with laminin deemed optimal for increasing outgrowth length, and netrin 1 for growth cone extension (Fligor et al., 2018). These phenotypes were achieved only when organoids were dissected and uniformly-sized aggregates were adhered in ECM substrates, meaning these guidance cues and resultant neurite growth are not facilitated within the whole retinal organoid suspension culture. However, laminin is expressed in retinal organoids (Dorgau et al., 2018). Laminin subtypes (α, β and γ chain) exhibit temporal-spatial expression patterns during retinogenesis (Byström et al., 2006; Libby et al., 2000). Blocking laminin γ3 function in developing retinal organoids leads to reduced expression of RGC markers *HUC* and *HUD*, and increased expression of the apoptosis marker caspase 3 (Dorgau et al., 2018). Thus, although some molecular cues essential for RGC development may be absent from organoid cultures, key signals such as laminin are functioning in the 3D environment.

The lack of neurotropic factors *in vitro* could be attributed to the loss of RGCs, possibly due to the central location of RGCs within the inner retinal layers of 3D retinal organoids. As such, a 2D environment may enable easier access to nutrients. However, this is an overly simplistic model. The absence of structures such as lens and surface ectoderm, as well as RGC dendrite connections with their target location in the visual cortex, may contribute to and enhance this phenotype. scRNAseq studies of 2D hPSC-derived RGCs has delineated distinct RGC subtypes, with one subtype exhibiting enriched axon guidance genes (Daniszewski et al., 2018). However, RGCs isolated from retinal organoid cultures contain a diverse RGC expression profile and divergent expression of guidance receptor genes (Fligor et al., 2018). Comparative analysis of 2D (Daniszewski et al., 2018), 3D-enriched (Fligor et al., 2018) and retinal organoid (Table 1) transcriptomic data will better inform the nature of RGC subtypes generated in organoids, while proteomics could highlight missing components that may support RGC development and survival.

**Table 1.**
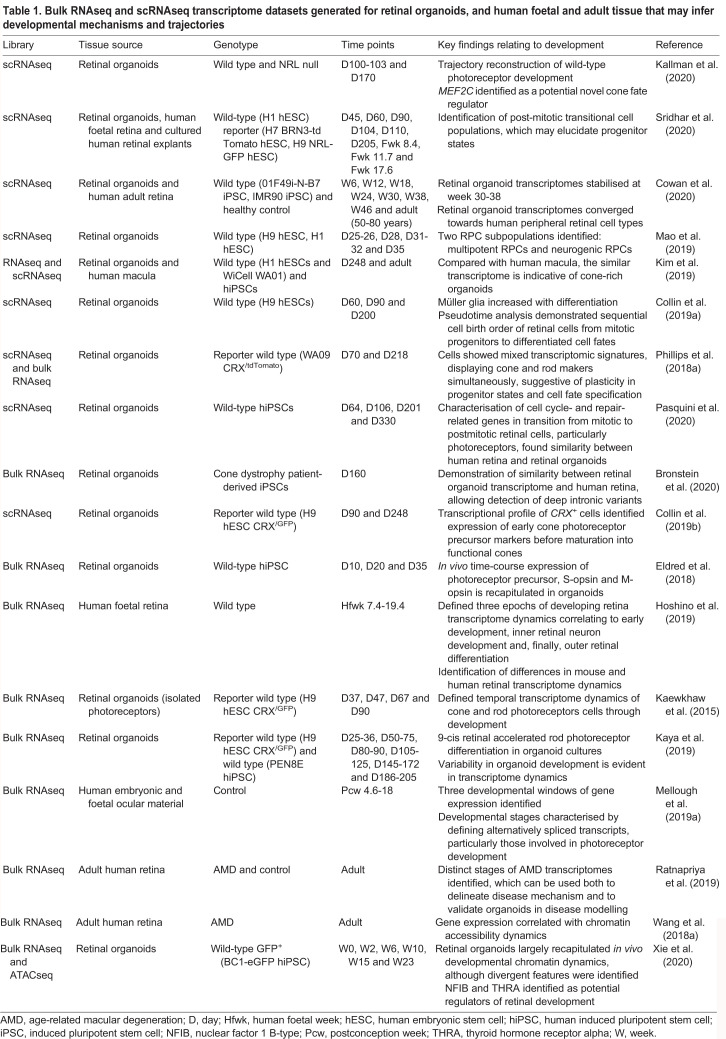

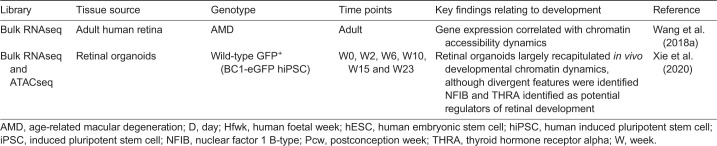
**Bulk RNAseq and scRNAseq transcriptome datasets generated for retinal organoids, and human foetal and adult tissue that may infer developmental mechanisms and trajectories**

### The overlooked interneurons and Müller glia

Whereas the development of RGCs and photoreceptor cells has been well characterised in retinal organoids, that of interneurons is yet to be investigated in detail. Transcriptome analysis has begun to delineate interneuron populations in organoids. The bipolar cell markers VSX1 and GRM6, which can be detected via RNAseq, are upregulated until month 9 of culture *in vitro* (Kim et al., 2019). Amacrine cells are the most diverse retinal cell, with >30 identifiable subtypes (Haverkamp and Wässle, 2000; MacNeil and Masland, 1998; MacNeil et al., 1999). Although largely elusive, the complex mechanisms of subtype specification can be partly attributed to temporal regulation (Cherry et al., 2009) and amacrine cell transcriptomes are accordingly dynamic during development (Kunzevitzky et al., 2010). In retinal organoids, although amacrine cells are detectable by immunohistochemistry, these are often not discreetly clustered in single-cell transcriptomic analyses (Kim et al., 2019). The rarity of bipolar, horizontal and amacrine cells in organoids poses a challenge for identifying them in cell-clustering analyses, highlighting the need for comparative cross-analysis of currently available datasets. In long-term culture, retinal organoids lose inner layer lamination, where these cells reside (Sridhar et al., 2020). Loss of synaptic partner RGCs and the resultant trophic deprivation and cell death (Fig. 4B) may also account for some of this disorganisation.

Müller glia, however, survive well in hPSC-derived cultures, displaying their typical morphology that spans the entire NR (Capowski et al., 2019; Mellough et al., 2019a,b; Slembrouck-Brec et al., 2019). Specific markers, including cellular retinaldehyde binding protein (*CRALBP*) and vimentin*,* increase in expression from days 90-200 (Collin et al., 2019a; Eastlake et al., 2019). Interestingly, scRNAseq finds that day 90 retinal organoid-derived Müller glia and photoreceptors cluster, with shared transcriptional profiles (Collin et al., 2019a). A separate scRNAseq study revealed a large number of cells identified as Müller glia in late-staged organoids (>26 weeks) (Sridhar et al., 2020). These studies demonstrated remarkable similarities in cell type proportions between organoids and the foetal retina of the equivalent stage, although differences in gene expression are observed in individual cell types.

### Human photoreceptor development in the single-cell context

Human and animal models display overt differences in ocular morphology. In nocturnal dichromats, such as the mouse, rod photoreceptors make up >70% of the retina (Blackshaw et al., 2001). In the trichromatic human and non-human primate, three cone photoreceptor subtypes facilitate maximal response to long, medium and short wavelengths (Nathans et al., 1986). Therefore, understanding progenitor cell fate choices towards rod photoreceptors and cone subtypes is particularly important in the context of human retinogenesis. The formation of the phototransduction machinery requires a complex cascade of gene regulatory networks. Retinal organoids provide a means of generating large-scale cultures, which are amenable to genetic manipulation (Box 1) and transcriptomic analysis, to infer novel mechanisms that govern photoreceptor development and maturation (Eldred et al., 2018; Kallman et al., 2020; Kim et al., 2019; Xie et al., 2020).

**Box 1. Gene editing of pluripotent stem cells lines as a tool for studying retinal development and disease**

Genome editing technology, including CRISPR-Cas9 and zinc-finger nucleases (ZFNs) have been used to generate mouse and human pluripotent stem cells (PSC) lines containing endogenous reporters and to introduce or correct disease specific mutations. Reporter lines have enabled the visualisation of specific cell types in both 2D and 3D differentiation cultures, which can be used to model development. A number of cell lines expressing earlier markers involved in retinal development have been generated (Lam et al., 2017, 2020; Sluch et al., 2018; Wu et al., 2018). A mouse *Rax.GFP* embryonic stem cell (ESC) line has been used in studies to discern both rostral hypothalamic and retinal progenitor cells (Wataya et al., 2008). This line was also used in the landmark demonstration of PSC-derived optic cup formation (Eiraku et al., 2011), before also being used to optimise retinal specification in SFEB cultures and isolate a pure population of retinal progenitors for further maturation (West et al., 2012). A human *BRN3B.tdTomato* ESC line, reporting a retinal ganglion cell (RGC)-specific homeodomain protein, was used to improve adherent differentiation to the RGC lineage (Sluch et al., 2015, 2017). Numerous human photoreceptor-specific cell lines have been established, with *CRX* being the most popular reporter gene as it enables the characterisation of photoreceptor genesis and isolation of photoreceptor precursors (Collin et al., 2016, 2019b; Kaewkhaw et al., 2015; Kaya et al., 2019; Phillips et al., 2018b). However, isolation of photoreceptor cells using CD protein surfaces markers has also been described (Gagliardi et al., 2018; Welby et al., 2017). Recently, genome-edited cell lines have been used to generate models of retinal disease in the dish (Capowski et al., 2014; Zheng et al., 2020). Patient-derived iPSC lines with disease-causing mutations have been corrected to create ideal isogenic control lines that can be differentiated in parallel to validate disease phenotypes (Lam et al., 2020; Lane et al., 2020; VanderWall et al., 2020). Edited cell lines are also valuable to test novel regulators of development and disease (Buskin et al., 2018; Deng et al., 2018; Eldred et al., 2018; Phillips et al., 2014).

In the past, studying human eye development was limited mostly to immunocytochemistry of a few markers. Cone-rod homeobox protein (CRX) is detectable in human foetal week (Fwk) 10.5 retinal tissue, becoming organised within a recognisable ONL framework by Fwk 14-15 (Bibb et al., 2001; O'Brien et al., 2003). In organoid cultures, CRX is present by 5-6 weeks, before gradually increasing in expression at the presumptive ONL by weeks 13-14 (Gonzalez-Cordero et al., 2017; Kuwahara et al., 2015; Mellough et al., 2015; Meyer et al., 2011; Reichman et al., 2014; Singh et al., 2015). Representing early post-mitotic photoreceptor precursors, this population is consolidated with the appearance and colocalisation of recoverin shortly thereafter (Gonzalez-Cordero et al., 2017; Mellough et al., 2015; Reichman et al., 2014; Singh et al., 2015; Wahlin et al., 2017).

Whereas these studies have provided insights into the differentiation of photoreceptors, a comparison of the transcriptome of human foetal and adult retina with that of retinal organoids has revealed the extent to which retinogenesis is recapitulated *in vitro* (Table 1). RNAseq analysis of retinal organoids has identified molecular signatures associated with photoreceptor development in hESC-derived 3D retina (Kaewkhaw et al., 2015). Matching immunohistochemistry results and transcriptome data revealed parallel trajectories to *in vivo* retinal differentiation, and common and distinctive features between humans and rodents. These molecular signatures defined the pathways underlying human photoreceptor development (Kaewkhaw et al., 2015).

The advent of scRNAseq transcriptome analysis has enabled the exploration of retinogenesis with unprecedented resolution. Several analytical methods have been developed for reconstructing developmental trajectories and pseudotime relationships (the position of the cell along a time trajectory) (discussed by Hie et al., 2020). These methods enable the study of cell developmental lineages and their transition between different cell states. A recent scRNAseq comparison of human foetal and retinal organoid tissue eliminated culturing artefacts by growing human retinal explants and retinal organoids in near-identical conditions (Sridhar et al., 2020). This comparison confirmed very similar ONL cellular compositions and identified a population of precursor cells transitioning from RPCs to photoreceptors, with defined gene expression profiles (Sridhar et al., 2020). Another scRNAseq study assembled informative trajectories of retinal organoid-derived photoreceptor development (Kallman et al., 2020). Developmental pseudotime trajectory analysis identified 590 differentially expressed genes in retinal organoids at the stage of rod versus cone specification, indicating the elaborate decisions involved in photoreceptor differentiation (Kallman et al., 2020). Moreover, these studies highlight intrinsic differences in murine and human retinogenesis, particularly regarding the complexity of the short (S)-wave (blue), medium (M)-wave (green) and long (L)-wave (red) cone subclass trichromatic mosaic arrangement (Eldred et al., 2018; Kallman et al., 2020). Retinal organoids recapitulate *in vivo* photoreceptor developmental dynamics, with temporal expression of S-opsin-positive cones, followed by onset of L/M-opsin expressing cones after a 20 day developmental delay, analogous to the foetal retina (Eldred et al., 2018). The fate decision between rod and cone photoreceptor cells is largely determined by neural retina leucine zipper (NRL), via nuclear receptor subfamily 2 group E member 3 (NR2E3), which in turn represses cone-specific genes (Chen et al., 2005; Mears et al., 2001). Mutations in *NRL* or *NR2E3* may clinically present as enhanced S-cone syndrome: a disproportional ratio of S:L/M cones (Wright et al., 2004). In line with the *in vivo* phenotype, retinal organoids derived from a homozygous null *NRL* patient also show dominance of S-cone cells (Kallman et al., 2020; Wright et al., 2004). Overall, these observations and datasets have pinpointed molecular signatures and networks involved in RPC-photoreceptor cell fate decisions in the developing human retina.

## The development of functional light-sensing structures in retinal organoids

### Macula *in vitro*: a realistic possibility?

**Fig. 5.**
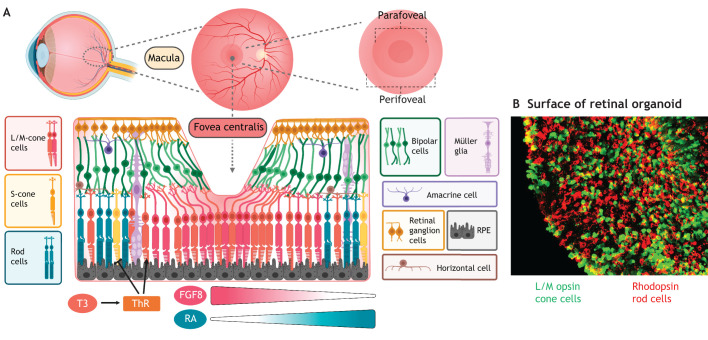
**Regionalisation of macula and photoreceptor cells in retinal organoids.** (A) *In vivo*, the central region of the human retina comprises the macula. At the centre of the macula, the fovea centralis is populated by long- (L) and medium- (M) wavelength cones, which are responsible for high acuity vision. The fovea centralis is flanked by para- and peri-foveal regions comprising both rod, and L/M and short- (S) cone photoreceptors. In retinal organoid cultures, a macula structure does not form, but candidate inducers may include thyroid hormone signalling via triiodothyronine (T3), which is involved in cone subtype specification, or modulation of retinoic acid (RA) and FGF8, which pattern the rod-free zone in the chick retina. (B) A 3D view of the surface of a 17-week-old retinal organoid shows photoreceptor subtypes, with rhodopsin^+^ rod photoreceptors (red) and L/M opsin^+^ cone photoreceptors (green). (B) Reproduced from Gonzalez-Cordero et al. (2017) where it was published under a CC-BY 4.0 license.

During development, retinogenesis initiates in the central retina with maturation occurring later in the periphery (Peters and Cepko, 2002; Young, 1985). This delayed peripheral differentiation supports the formation of the central cone-rich macula and perifoveal rod populations (Box 2). The macula, a ∼5 mm diameter anatomically specialised structure, is situated in the central region of the primate retina, with distinct photoreceptor cell populations and morphology. The fovea centralis (fovea), a central pit in the macula, represents a dense L/M cone population responsible for high visual acuity. The surrounding para- and perifoveal regions contain a mixed population of cone and rod photoreceptors (Fig. 5A). The precise timing and positional events leading to macular formation in humans cannot be satisfactorily uncovered using animal models, which do not have an equivalent structure. PSC-derived retinal organoids offer a human model that enables the study of macular development if this specialised area is formed *in vitro.* Most studies report generation of perifoveal-like NR from organoids with higher rod:cone ratios, and later stage organoid transcriptomes (>30 weeks) have been identified to strongly correlate with adult human peripheral retina (Capowski et al., 2019; Cowan et al., 2020; Gonzalez-Cordero et al., 2017). We have previously demonstrated the presence of RPCs in retinal organoids that are involved in cone fate specification, findings since corroborated by scRNAseq studies (Collin et al., 2019a; Gonzalez-Cordero et al., 2017; Kallman et al., 2020). Furthermore, cone-rich organoids have been described, with scRNAseq determining retinal organoid-derived cones to correlate more with macaque foveal cones than with peripheral-located cones (Kim et al., 2019; Peng et al., 2019). However, immunohistochemistry studies fail to demonstrate the typical macular regional specification: L/M-cones residing in the fovea are consistently absent, and rod and cone subtypes are instead located throughout the ONL (Fig. 5B).

**Box 2. Central-to-peripheral retinogenesis**

During retinal organoid differentiation, retinal progenitor cells (RPCs) spontaneously differentiate and migrate in a central to peripheral wave, mimicking *in vivo* retinogenesis. Downregulation of neurogenic RPC markers along a central-peripheral gradient is identifiable in retinal organoids, whereas multipotent RPCs reside peripherally, and β-catenin expression concurrently decreases along the peripheral-central gradient (Mao et al., 2019). Blimp1, which functions in normal development to inhibit re-specification of photoreceptors into RGCs, emerges centrally in organoid-derived neural retina before expanding to the periphery (Mao et al., 2019). Combined with the temporal specification of cones before rods, this is indicative of some preferential positioning of photoreceptor precursors prior to the stage of fate commitment, which has the potential to pattern a cone-rich region *in vitro*.

Further manipulation of developing organoids *in vitro* is needed to generate a macular region. Exploiting thyroid hormone (TH) signalling, which is involved in cone subtype specification, could be conducive to generating regionalised cone-rich NR (Eldred et al., 2018). Once committed to a cone fate, specification of cone precursors to M- and S-subtypes is mediated by thyroid hormone receptor beta (THRβ) (Glaschke et al., 2011; Ng et al., 2001; Roberts et al., 2006). Treatment of wild-type organoids throughout the developmental windows of photoreceptor birth and maturation with active TH triiodothyronine (T3) results in a significant conversion of S- to M-opsin^+^ cones (Eldred et al., 2018). Given that knockout of *Thrb* splice isoform *Thrb2* in the mouse results in the development of S-opsin cones and no M-opsin, *THRB2* was assumed to be a critical regulator of cone subtype specification in humans (Ng et al., 2001). However, CRISPR/Cas9-mediated knockout of *THRB2* in human retinal organoids did not alter the ratio of S:L/M-cone photoreceptors, suggesting cone fate determination in human retinogenesis is under more complex control (Eldred et al., 2018).

In the chick, creation of a rod-free zone of high visual acuity requires focal regulation of RA and FGF8 (da Silva and Cepko, 2017). Thus, animal models may provide further insights into the signalling networks patterning these specialised structures. Ultimately, a detailed computational meta-analysis of the available transcriptomic datasets will uncover more nuances in development between human foetal samples and organoids derived by different differentiation protocols. This may identify molecular cues currently absent *in vitro* that are essential for forming a macula – a vital next goal in advancing organoid technology.

### Are photoreceptor cells in retinal organoids mature and functional?

**Fig. 6.**
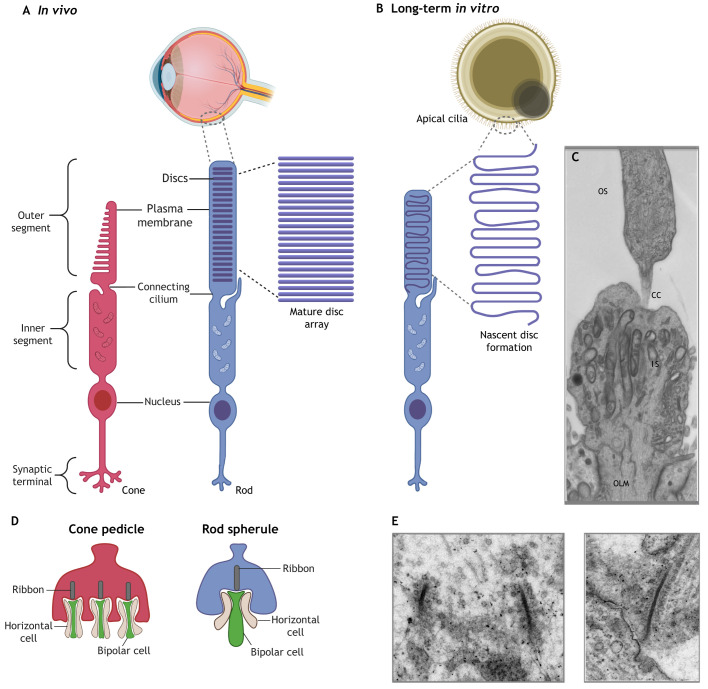
**Formation of mature photoreceptor structures.** (A) *In vivo*, cone and rod photoreceptors form a mature inner segment (IS), connecting cilia (CC) and outer segment (OS) in organised disc arrays, and ribbon synapses at their end-feet. (B,C) Photoreceptor cells in retinal organoids form similar IS, CC and OS-like structures with nascent discs (B), identifiable via electron microscopy as structures located apically to the elongated photoreceptor cilium (C). (D) Cone pedicles, the synaptic terminals of cone photoreceptors, form tripartite synapses with horizontal and bipolar cell dendrites. Rod synaptic terminals, the rod spherule, form a single ribbon synapse with horizontal and bipolar cells. (E) In retinal organoids, electron microscopy shows electron-dense ribbon synapses surrounded by synaptic vesicles. (C) Reproduced from Ovando-Roche et al. (2018) where it was published under a CC-BY 4.0 license. (E) Reproduced from Gonzalez-Cordero et al. (2017) where it was published under a CC-BY 4.0 license.

Photoreceptor maturation entails the generation of highly specialised outer segment structures responsible for light detection, the phototransduction cascade and the formation of synaptic connections (Fig. 6A). Earlier 2D and 3D differentiation protocols reported low numbers of photoreceptor cells, as they lacked visible outer segment-like structures both in mouse and human PSCs, suggesting *in vitro* conditions failed to recapitulate the complex developmental niches demanded by photoreceptor maturation (Kuwahara et al., 2015). However, advanced maturation conditions may now generate nascent apical cilia-like structures with a marked increase in rhodopsin localisation and outer segments with developing disc morphology (Gonzalez-Cordero et al., 2017; Lowe et al., 2016) (Fig. 6B,C). Time-dependent addition of RA (during weeks 10-14, a period of photoreceptor specification) to retinal organoids also increased rhodopsin expression, emulating the dynamic response to RA signalling in photoreceptor cells observed during zebrafish retinogenesis (Prabhudesai et al., 2005; Stevens et al., 2011; Zhong et al., 2014). Conversely, optimal conditions for cone photoreceptor development may require downregulation of RA, as seen in chick and mouse PSC-retinal organoids (Kruczek et al., 2017; da Silva and Cepko, 2017). Finally, mature photoreceptor formation has also been demonstrated in the absence of RA supplementation (Li et al., 2018). As such, the important role of RA in photoreceptor specification and maturation requires further characterisation *in vitro*.

Transcriptome analysis of retinal organoids suggest they reach a stable development state by week 30-38 (Cowan et al., 2020). At this stage, retinal organoids model *in vivo* development more precisely, supporting the formation of mature structures and synaptogenesis. Outer segment-like structures in retinal organoids are reported to grow to 39 µm terminal length, mimicking *in vivo* development (Wahlin et al., 2017). Comparative transcriptome analysis between mouse foetal and mPSC-derived retinal organoids identified components lacking *in vitro*, i.e. docosahexaenoic acid (DHA) and FGF1, addition of which facilitated enhanced photoreceptor maturation, and could also be applied to hPSC-derived cultures (Brooks et al., 2019). Calcium influx assays and light response patch-clamping studies have demonstrated some functionality of photoreceptors in hPSC-derived retinal organoids, but further functional investigation is required (Cowan et al., 2020; Gagliardi et al., 2018; Mellough et al., 2015; Reichman et al., 2017; Zhong et al., 2014).

The synaptic terminals of photoreceptors, the cone pedicle and rod spherule sit at the ONL-OPL border as soma enlargements or extensions. Releasing inhibitory glutamate-filled synaptic vesicles while depolarised in the dark, following light excitation, pedicles and spherules relay the light signal onto bipolar and horizontal cell dendrites. Important indicators of mature photoreceptors – typical ribbon synapses in close proximity to synaptic vesicles – are identifiable as electron-dense bars using ultra-structure microscopy in retinal organoid cultures (Fig. 6D,E) (Cora et al., 2019; Gonzalez-Cordero et al., 2017; Wahlin et al., 2017). Localisation of the presynaptic protein bassoon or the photoreceptor presynaptic terminal scaffolding protein post-synaptic density-95 (PSD95) has been described in juxtaposition to ribbon marker C-terminal binding protein (CTBP2) or its isoform ribeye (Cora et al., 2019; Gonzalez-Cordero et al., 2017; Koulen et al., 1998; Mellough et al., 2015; Ovando-Roche et al., 2018; Wahlin et al., 2017). Advanced culture conditions utilising spinning bioreactors reported increased photoreceptor yields and ribeye (*CTPB2*) expression in the OPL by week 16 (Ovando-Roche et al., 2018). More recently, high-resolution light sheet imaging visualised ribbon synapse networks in week 41 whole-retinal organoids, resolving single cells within a preserved 3D spatial morphology (Cora et al., 2019). An optimised passive clarity technique, employing hydrogel-based clearing, visualised the ribeye^+^ site of the synapse between arrestin 3^+^ cone photoreceptors and PCKα^+^ bipolar cells (Cora et al., 2019). The postsynaptic marker vesicular glutamate transporter 1 (VGLUT1) has been shown to colocalise at putative photoreceptor terminals, and syntaxin can be found both directly and further basal to the ONL, demarcating a presumptive IPL and OPL (Dorgau et al., 2019; Hallam et al., 2018; Sridhar et al., 2020). However, these postsynaptic markers are less well characterised and elusive in long-term cultures. Further implementation of such high-resolution imaging techniques at earlier time points, combined with detailed transcriptomic analysis, will provide more insight into the mechanisms of both outer segment maturation and synaptogenesis.

## Next-generation retinal organoids

Significant advance has been made in the morphological and molecular characterisation of human retinal organoids and the demonstration of their utility in understanding the development of the human retina and its diseases. Studies have divulged RPC trajectories and cell fate decisions in early retinal development (Table 1). Specifically, intermediate progenitors for various retinal cell types, particularly cone photoreceptors, have been identified (Collin et al., 2019a; Eldred et al., 2018; Phillips et al., 2018a; Sridhar et al., 2020). However, notwithstanding the progress, the field is still in its infancy with several limitations to overcome.

Studies note not only temporal and cellular variability of retinal organoids derived by different protocols, but also from different iPSC lines (Capowski et al., 2019; Chichagova et al., 2020; Kaya et al., 2019; Mellough et al., 2019a; Wang et al., 2018b). This may be attributed to epigenetic memory: an intrinsic shortcoming of reprogrammed hPSCs. Compilation of transcriptomic datasets will be key in defining more robust and global profiles at each developmental time-point, allowing better definition of protocol standards. Another challenge is in the scalability and laborious nature of the differentiation process. However, new methods to easily isolate hPSC-derived optics vesicles are beginning to be explored (Regent et al., 2020).

Thus, the initial choice of a robust and reliable protocol is important, as is the ability to screen and reduce in/between-batch variability through selection of discernible morphological features. In 2D/3D approaches, retinal vesicles arise within RPE islands, enabling precise isolation over forebrain organoids also present in the culture. In aggregation-based 3D cultures, contamination of brain-derivatives creates variability in downstream experiments. Phenotypic alterations that appear during the culture process could be easily discerned using computational or bioinformatic methodology, such as machine-learning methods, and integration of omics and imaging. Studies have begun to predict the differentiation efficiency of retinal organoid cultures based on bright-field images (Kegeles et al., 2020) or functionality and quality of hPSC-derived RPE from live or immunofluorescence images (Schaub et al., 2020; Ye et al., 2020).

Current retinal organoid models lack the complex organisation of ocular with non-ocular tissues. Some studies claim formation of surface-ectoderm derivatives, rudimentary lens and corneal tissue or whole-corneal organoids but, to date, these structures have not been generated in a single construct (Foster et al., 2017; Mellough et al., 2015, 2019b). Formation and invagination of surface ectoderm *in vitro* would best recapitulate inductive signals in early retinogenesis, and could be explored with manipulation of FGF or migration of periocular mesenchyme (Hyer et al., 2003; Llonch et al., 2018). Potentially crucial to the functionality of these models is the precise apposition of RPE with photoreceptors, RGC/interneuron survival and the formation of a macula-like region.

**Fig. 7.**
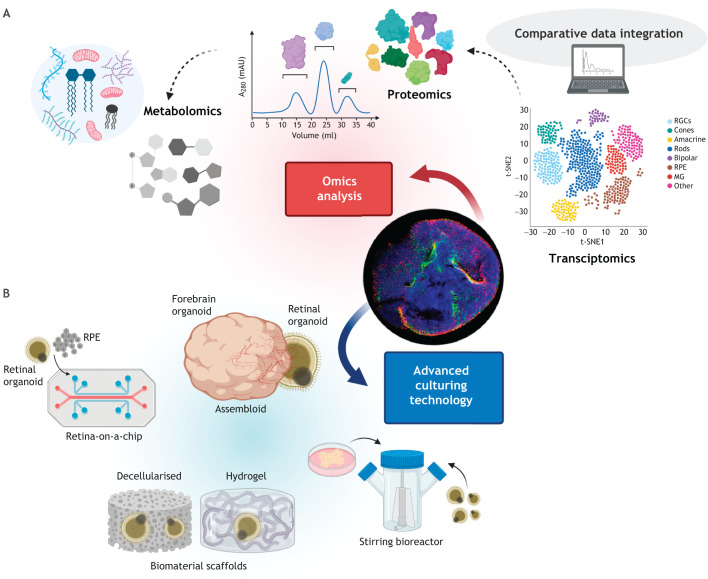
**The future of retinal organoids: functional study and advanced culture systems.** Organoids facilitate both omics studies and the development of complex mini organs in the dish. (A) Computational data analysis of transcriptomic datasets of retinal organoids will uncover novel developmental cell characteristics and validate differentiation protocols, whereas downstream proteomic and metabolomic studies will help to inform functionality. (B) Advanced culturing systems, including biomaterials and scaffolds to maintain a 3D niche, bioreactors to improve aeration and organ-on-a-chip approaches to incorporate vasculature, should be considered to improve differentiation and maturation, whereas co-culture of brain and retinal organoids (assembloid technology) may generate appropriate neuroretina-brain connections. The central image shows a retinal organoid expressing rhodopsin^+^ rod photoreceptors (red) and L/M opsin^+^ cone photoreceptors (green) (reproduced from Gonzalez-Cordero et al., 2017 where it was published under a CC-BY 4.0 license). MG, Müller glia; RGCs, retinal ganglion cells; RPE, retinal pigment epithelium.

Although comparison of omics datasets (Fig. 7A) will go some way to infer functionality, validation via electrophysiology *in vitro* or visual rescue following cell transplantation *in vivo* is ultimately required. Light-driven electrophysiological responses of hPSC-derived retinal organoids are reportedly immature, resembling those in the neonatal mouse (Hallam et al., 2018). In normal development, maturation and refinement of retinal synapses persists postnatally in response to activity (Wang et al., 2001). To model this *in vitro*, maturation and persistence of RGCs and interneurons must be attained. Bioreactor cultures have been demonstrated to improve laminar stratification and increase formation of complex structures, due to improved aeration and nutrient distribution (DiStefano et al., 2018; Ovando-Roche et al., 2018). However, these cultures are still imperfect and emerging technologies in biomaterials, scaffolds, de-cellularisation and vascularisation may encourage cell survival and longer-term maintenance of lamination (Achberger et al., 2019; Chen et al., 2019; DiStefano et al., 2018; Dorgau et al., 2019; Hertz et al., 2013; Wörsdörfer et al., 2019). *In vivo*, the lens and ciliary body biosynthesise ECM components that may act as guidance cues for RGC outgrowth. Encouraging the *in vitro* generation of these other eye structures may promote RGC maturation (Halfter et al., 2005). Organ-on-a-chip technology merges cell biology and bioengineering, creating a biomimetic micro-physiological system on a perfusion microfluidic chip that mimics vasculature circuitry (Huh et al., 2010). The advent of retina-on-a-chip may begin to address the above shortcomings of organoid cell culture (Achberger et al., 2019). However, these systems have not yet been demonstrated to persist in long-term culture and require more specialised platforms, exceeding the remarkable simplicity of self-organising protocols.

Finally, the generation of assembloids promises to improve organoid development and maturation. Vascularisation and generation of stromal components in neural organoids has been achieved through co-culture with mesodermal progenitor cells (Wörsdörfer et al., 2019). The fusion of brain-region specific organoids to create forebrain assembloids capable of modelling *in vivo* neuronal interactions has also been described (Bagley et al., 2017; Sloan et al., 2018) (Fig. 7B). A compelling challenge in retinal and ocular organoid modelling is the integration of bioengineering and assembloid methodology to generate long-term hybrid forebrain organoids, comprising eye cups with polarised mature ocular structures and functional neuronal circuitry. Such complex assembloids would better elucidate the dynamics of retinogenesis during human eye development.

## Conclusion: the importance of robust retinal models

The requisite of well-characterised models is essential to fully understand how well current models are mimicking normal development. Retinal organoids represent unique platforms for modelling human disease, therapies (reviewed by Kruczek and Swaroop, 2020) and development, but studies must take into account that disease phenotypes might be an experimental artefact due to artificial culture conditions. As a human model, the expression of key ECM components and cell-surface markers are recapitulated in hPSC-derived retinal organoids more faithfully than in animal models (Felemban et al., 2018). Chromatin accessibility dynamics and mRNA splicing programmes have been largely found to imitate human foetal and adult samples (Kim et al., 2019; Xie et al., 2020). Other omics studies, such as proteome analysis alongside metabolomics, are crucial for corroborating gene expression data and inferring functionality. This will enable the design of assays to identify robust disease-relevant biomarkers and the development of new therapeutic approaches, such as gene and cell therapies. Gene therapy in the eye has pioneered this field of research with murine models providing proof of concept for numerous studies reaching clinical trials (reviewed by Trapani and Auricchio, 2018). PSC-derived disease-specific retinal organoids could now be used to demonstrate efficacy for new gene therapies. Enthusiasm in the field currently surrounds moving towards clinical trials for organoid-derived photoreceptor cell therapy. Multiple groups have demonstrated isolation and functional integration of hPSC-derived photoreceptor cells *in vivo* (reviewed by Gasparini et al., 2019). To substantiate and improve this regenerative medicine approach, it is crucial to delineate the unknown intricacies of human retinal development and how faithfully we are truly recapitulating this process in organoid systems.
